# Can Machine Learning Approaches Lead Toward Personalized Cognitive Training?

**DOI:** 10.3389/fnbeh.2019.00064

**Published:** 2019-04-04

**Authors:** Reut Shani, Shachaf Tal, Sigal Zilcha-Mano, Hadas Okon-Singer

**Affiliations:** ^1^Department of Psychology, University of Haifa, Haifa, Israel; ^2^The Integrated Brain and Behavior Research Center (IBBR), University of Haifa, Haifa, Israel

**Keywords:** cognitive training, cognitive remediation, machine learning, treatment adaptation, treatment selection

## Introduction

Cognitive training efficacy is controversial. Although many recent studies indicate that cognitive training shows merit, others fail to demonstrate its efficacy. These inconsistent findings may at least partly result from differences in individuals' ability to benefit from cognitive training in general, and from specific training types in particular. Consistent with the move toward personalized medicine, we propose using machine learning approaches to help optimize cognitive training gains.

## Cognitive Training: State-of-the-Art Findings and Debates

Cognitive training targets neurobiological mechanisms underlying emotional and cognitive functions. Indeed, Siegle et al. (2007) suggested that cognitive training can significantly improve mood, daily functioning, and cognitive domains. In recent years, various types of cognitive training have been researched. Frequently researched training types include *cognitive bias modification* (CBM) aims to modify cognitive processes such as interpretations and attention, making these more adaptive and accommodating to real-life demands (Hallion and Ruscio, 2011); *inhibitory training* seeks to improve inhibitory control and other executive processes, thus helping regulate behavior and emotion (Cohen et al., 2016; Koster et al., 2017); *working memory training* targets attentional resources, seeking to increase cognitive abilities by improving working memory capacities (Melby-Lervåg and Hulme, 2013). All these types demonstrated major potential in improving psychopathological symptoms or enhancing cognitive functions (Jaeggi et al., 2008; Hakamata et al., 2010).

Despite the accumulating body of evidence suggesting that cognitive training is a promising research path with major clinical potential, questions remain regarding its efficacy, and generalizability. Recent meta-analyses further corroborate this (for a discussion, see Mogg et al., 2017; Okon-Singer, 2018). For example, several research groups tested CBM studies using meta-analyses. Hakamata et al. (2010) analyzed twelve studies (comprising 467 participants from an anxious population), reporting positive moderate effects of training on anxiety symptom improvement. Yet two other meta-analyses focusing on both anxiety and depression (49 and 45 studies, respectively) demonstrated small effect sizes and warned of possible publication bias (Hallion and Ruscio, 2011; Cristea et al., 2015). These inconsistent results raise important questions about training efficacy. Several factors have been suggested as potential sources of this variability in effect size, including differences in inclusion criteria and quality of the studiesincluded (Cristea et al., 2015).

As in the CBM literature, meta-analyses of working memory training also yielded divergent results. Au et al. (2015) analyzed twenty working memory training studies comprising samples of healthy adults and reported small positive effects of training on fluid intelligence. The authors suggested that the small effect size underestimates the actual training benefits and may result from methodological shortcomings and sample characteristics, stating that “it is becoming very clear to us that training on working memory with the goal of trying to increase fluid intelligence holds much promise” (p. 375). Yet two other meta-analyses of working memory (87 and 47 studies, respectively) described specific improvements only in the trained domain (i.e., near transfer benefits) and few generalization effects in other cognitive domains (Schwaighofer et al., 2015; Melby-Lervåg et al., 2016). As with CBM, these investigations did not include exactly the same set of studies, making it difficult to infer the reason for the discrepancies. Nevertheless, potential factors contributing to variability in intervention efficacy include differences in methodology and inclusion criteria (Melby-Lervåg et al., 2016).

Some scholars suggested that the inconsistent results seen across types of training may be result from the high variability in training features, such as dose, design type, training type, and type of control groups (Karbach and Verhaeghen, 2014). For example, some studies suggest that only active control groups should be used and that using untreated controls is futile (Melby-Lervåg et al., 2016), while others discovered no significant difference between active and passive control groups (Schwaighofer et al., 2015; Weicker et al., 2016). Researchers have also suggested that the type of activity assigned to the active control group (e.g., adaptive or non-adaptive) may influence effect sizes (Weicker et al., 2016). Adaptive control activity may lead to underestimation of training benefits, while non-adaptive control activity may yield overestimation (von Bastian and Oberauer, 2014).

Training duration has also been raised as a potential source of variability. Weicker et al. (2016) suggested that the number of training sessions (but not overall training hours) is positively related to training efficacy in a brain injured sample. While only studies with more than 20 sessions demonstrated a long-lasting effect. In a highly influential working memory paper, Jaeggi et al. (2008) compared different numbers of training sessions (8–19). Outcomes demonstrated a dose-dependency effect: the more training sessions participants completed, the greater the “far transfer” improvements. In contrast, in a 2014 meta-analytical review Karbach and Verhaeghen reported no dose–dependency, as overall training time did not predict training effects. This is somewhat consistent with the findings of Lampit et al. (2014) meta-analysis, which indicated that only three or fewer training sessions per week were beneficial in training healthy older adults in different types of cognitive tasks. Furthermore, even time gaps between training sessions when the overall number of sessions is fixed may be influential. A study that specifically tested the optimal intensity level of working memory training revealed that distributed training (16 sessions in 8 weeks) was more beneficial than high intensity training (16 sessions in 4 weeks) (Penner et al., 2012). In sum, literature reviews maintain that this large variability in training hampers attempts to evaluate the findings (Koster et al., 2017; Mogg et al., 2017).

So far, the majority of studies in the field of cognitive training have been concerned mainly with establishing the average effectiveness of various training methods, with studies based on combined samples comprising individuals who profited from training and those who did not. Therefore, the samples' heterogeneity might be too high to evaluate efficacy for the “average individual” in each sample. We contend that focusing on the average individual contributes to the inconsistent findings, as is also the case with other interventions aimed at improving mental health (Zilcha-Mano, 2018). We argue that the inconsistent findings and large heterogeneity in studies evaluating cognitive training efficacy do not constitute interfering noise but rather provide important information that can guide us in *training selection*. In addition to selecting the optimal training for each individual, achieving maximum efficacy also requires adapting the selected training to each individual's characteristics and needs (Zilcha-Mano, 2018). In line with this notion, training games studies (i.e., online training platforms displayed in a game-like format) showcased different methods which personalized cognitive training by (a) *selecting* the type of training according to a baseline cognitive strengths and weaknesses evaluation or the intent of the trainee, and (b) *adapting* the ongoing training according to the individual's performance (Shatil et al., 2010; Peretz et al., 2011; Hardy et al., 2015). Until now, however, training personalization was made by pre-exist defined criteria and rationale (i.e., individual's weaknesses and strengths, individual's personal preference). Additional method for personalization, that is becoming increasingly popular in recent years, is data-driven personalization implemented by machine learning algorithms (Cohen and DeRubeis, 2018).

The observed variation in efficacy found in cognitive training studies may serve as a rich source of information to facilitate both intervention selection and intervention adaptation—the two central approaches in personalized medicine (Cohen and DeRubeis, 2018). Intervention selection seeks to optimize intervention efficacy by identifying the most promising type of intervention for a given individual based on as many pre-training characteristics as possible (e.g., age, personality traits, cognitive abilities). Machine learning approaches are especially suitable for such identification because they enable us to choose the most critical items for guiding treatment selection without relying on specific theory or rationale. In searching for a single patient characteristic that guides training selection, most approaches treat all other variables as noise. It is more intuitive, however, to hypothesize that no single factor is as important in identifying the optimal training for an individual as a set of interrelated factors. Traditional approaches to subgroup analysis, which tests each factor as a separate hypothesis, can lead to erroneous conclusions due to multiple comparisons (inflated type I errors), model misspecification, and multicollinearity. Findings may also be affected by publication bias because statistically significant moderators have a better chance of being reported in the literature. Machine learning approaches make it feasible to identify the best set of patient characteristics to guide intervention and training selection (Cohen and DeRubeis, 2018; Zilcha-Mano et al., 2018). With that said, given the flexibility of methods like decision tree analyses, there is a risk of overfitting that reduces validity for inference out of sample, such that the model will fit specifically the sample on which it was built and may be therefore unlikely to be generalizable in an independent application (Ioannidis, 2005; Open Science Collaboration, 2015; Cohen and DeRubeis, 2018). Thus, it is important to test out-of-sample prediction, either on a different sample or a sub-sample of the original sample on which the model was not built (e.g., cross-validation).

An example of treatment selection from the field of antidepressant medication (ADM) demonstrates the utility of this approach. Current ADM treatments are ineffective for up to half the patients, despite much variability in patient response to treatments (Cipriani et al., 2018). Researchers are beginning to realize the benefits of implementing machine learning approaches in selecting the most effective treatment for each individual. Using the gradient boosting machine (GBM) approach, Chekroud et al. (2016) identified 25 variables as most important in predicting treatment efficacy and were able to improve treatment efficacy in 64% of responders to medication—a 14% increase.

**Figure 1:**
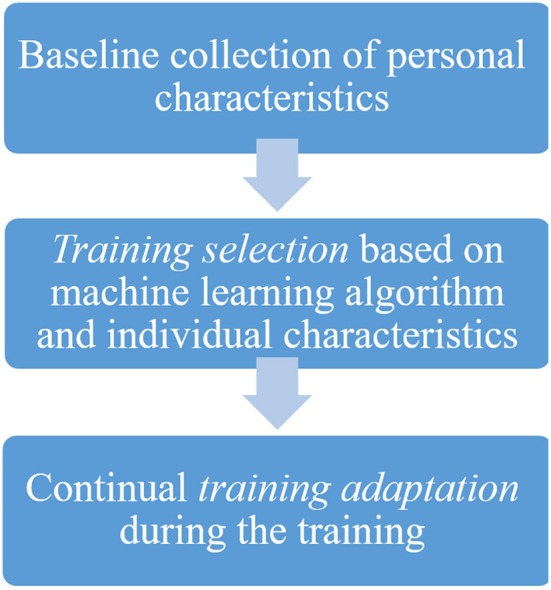
Flow diagram of personalized cognitive training process.

Whereas, *training selection* affects pre-treatment decision-making, *training adaptation* focuses on continuously adapting the training to the individual (see Figure 1). A patient's baseline characteristics (e.g., age, personality traits, cognitive abilities) and individual training performance trajectory can be used to tailor the training parameters (training type, time gaps between sessions, number of sessions, overall training hours) to achieve optimal performance. Collecting information from a sample of patients with similar baseline characteristics that underwent the same intervention yields an expected trajectory. Deviations from this expected trajectory act as warning signs and can help adapt the training parameters to the individual's needs (Rubel and Lutz, 2017).

An example of treatment adaptation comes from the field of psychotherapy research, where a common treatment adaptation method involves providing therapists with feedback on their patients' progress. This method was developed to address the problem that many therapists are not sufficiently aware of their patients' progress. While many believe they are able to identify when their patients are progressing as expected and when not, in practice this may not be true (Hannan et al., 2005). Many studies have demonstrated the utility of giving therapists feedback regarding their patients' progress (Lambert et al., 2001; Probst et al., 2014). Shimokawa et al. (2010) found that although some patients continue improving and benefitting from therapy (on-track patients—OT), others seem to deviate from this positive trajectory (not-on-track patients—NOT). These studies provided clinicians feedback on their patients' state so they could better adapt their therapy to the patients' needs. This in turn had a positive effect on treatment outcomes in general, especially outcomes for NOT patients, to the point of preventing treatment failure. These treatment adaptation methods have recently evolved to include implementations of the nearest neighbor machine learning approach originating in avalanche research (Brabec and Meister, 2001), as well as other similar approaches to better predict an individual's optimal trajectory and identify deviations from it (Rubel et al., in press).

Machine learning approaches may thus be beneficial in the efforts of progressing toward personalized cognitive training. The inconsistencies between studies in terms of the efficacy of CBM, inhibitory training, and working memory training can serve as a rich and varied source to guide the selection and adaptation of effective personalized cognitive training. In this way, general open questions such as optimal training duration and time gaps between sessions will be replaced with specific questions about the training parameters most effective for each individual.

## Author Contributions

RS managed the planning process of the manuscript, performed all administrative tasks required for submission and drafted the manuscript. HO-S and SZ-M took part in planning, supervision, brainstorming, and writing the manuscript. ST took part in brainstorming and writing the manuscript.

### Conflict of Interest Statement

The authors declare that the research was conducted in the absence of any commercial or financial relationships that could be construed as a potential conflict of interest.
